# Emerging Roles of Motile Epidermal Chloroplasts in Plant Immunity

**DOI:** 10.3390/ijms23074043

**Published:** 2022-04-06

**Authors:** Hiroki Irieda

**Affiliations:** Academic Assembly, Institute of Agriculture, Shinshu University, Nagano 399-4598, Japan; irieda@shinshu-u.ac.jp; Tel.: +81-265-77-1428

**Keywords:** epidermal chloroplast, chloroplast movement, stromule, plant pathogen, penetration, nonhost resistance, preinvasive defense, *Arabidopsis thaliana*, *Nicotiana benthamiana*, CHUP1

## Abstract

Plant epidermis contains atypical small chloroplasts. However, the physiological role of this organelle is unclear compared to that of large mesophyll chloroplasts, the well-known function of which is photosynthesis. Although knowledge of the involvement of chloroplasts in the plant immunity has been expanded to date, the differences between the epidermal and mesophyll chloroplasts are beyond the scope of this study. Given the role of the plant epidermis as a barrier to environmental stresses, including pathogen attacks, and the immune-related function of chloroplasts, plant defense research on epidermal chloroplasts is an emerging field. Recent studies have revealed the dynamic movements of epidermal chloroplasts in response to fungal and oomycete pathogens. Furthermore, epidermal chloroplast-associated proteins and cellular events that are tightly linked to epidermal resistance against pathogens have been reported. In this review, I have focused on the recent progress in epidermal chloroplast-mediated plant immunity.

## 1. Introduction

The epidermis of multicellular organisms, including plants, acts as a barrier to protect against a variety of stresses such as changes in the external environment and pathogen attacks. From the perspective of pathogens, breaking through the epidermal cells of the plant is an essential first step in invasion; in particular, fungal and oomycete pathogens need to penetrate directly into the plant epidermis and develop invasive hyphae inside for successful infection. For instance, the anthracnose fungi *Colletotrichum* species and rice blast fungus *Pyricularia oryzae* (syn. *Magnaporthe oryzae*) form a melanized dome-shaped cell called an appressorium on the plant surface to develop a penetration peg in the epidermis of the host plants, followed by invasive hyphal extension and outbreak of destructive disease [1]. However, if the plant is a nonhost, these fungal pathogens can form melanized appressorium, but cannot penetrate into the epidermal cell owing to the pre-invasive nonhost resistance (NHR) of plants, which generally provides durable, robust, and broad-spectrum immunity and effectively prevents the invasion of a vast number of nonadapted fungi and oomycetes in incompatible interactions [2,3]. In the model brassicaceous plant *Arabidopsis thaliana*, it has been reported that many immune pathways and components underpin the deployment of epidermal NHR in a multilayered manner against the entry of nonadapted fungal and oomycete pathogens, such as *Blumeria graminis* f. sp. *hordei* [4,5,6,7,8,9,10,11,12], *Colletotrichum tropicale* [13,14,15,16], *P. oryzae* [17,18,19,20,21,22], and *Phytophthora infestans* [6,7]. However, a complete understanding of the molecular basis of preinvasive NHR remains elusive. To gain a comprehensive understanding of NHR, it is important to identify unknown immune-related events and components in plant epidermis.

In higher plants, except few plants, such as tobacco, it was long believed that no chloroplasts exist in epidermal cells other than guard cells [23,24,25,26]. Most studies on chloroplast focus on mesophyll cells, where numerous typical large chloroplasts are highly differentiated for photosynthesis [27,28,29]. However, chlorophyll-containing atypical small chloroplasts have also been observed in the epidermal pavement cells of *A. thaliana*, although thylakoids, which are responsible for light-dependent photosynthesis reactions, are poorly developed [30,31,32,33,34,35,36]. Therefore, the physiological role of epidermal chloroplasts is an emerging topic in the field of plant science.

Chloroplasts are known to function as environmental sensors against multiple biotic and abiotic stresses [37,38]. For instance, for plant immune responses against pathogens, secondary messengers, such as reactive oxygen species (ROS) and calcium (Ca^2+^), and the precursors of phytohormones, such as salicylic acid (SA), jasmonic acid, and abscisic acid, are all derived from chloroplasts [39,40,41,42]. There are many excellent reviews on the roles of chloroplasts in plant immunity. I refer the readers to recent reviews, which will provide an overview of chloroplast-related plant defense responses [43,44,45,46,47].

However, there are very few plant defense studies that focus on epidermal chloroplasts, and the functional differences between epidermal and mesophyll chloroplasts in the plant immune system have not drawn much attention. Recently, some reports demonstrated the involvement of epidermal chloroplasts in plant immunity. In this review, the primary focus is on epidermal chloroplasts, and the recent progress in plant epidermal immunity has been described.

## 2. Epidermal Chloroplast Response Controls the Entry of Fungal Pathogens in *A. thaliana*

### 2.1. Intracellular Movements of Epidermal Chloroplasts in Response to Fungal Pathogens in A. thaliana

**Figure 1:**
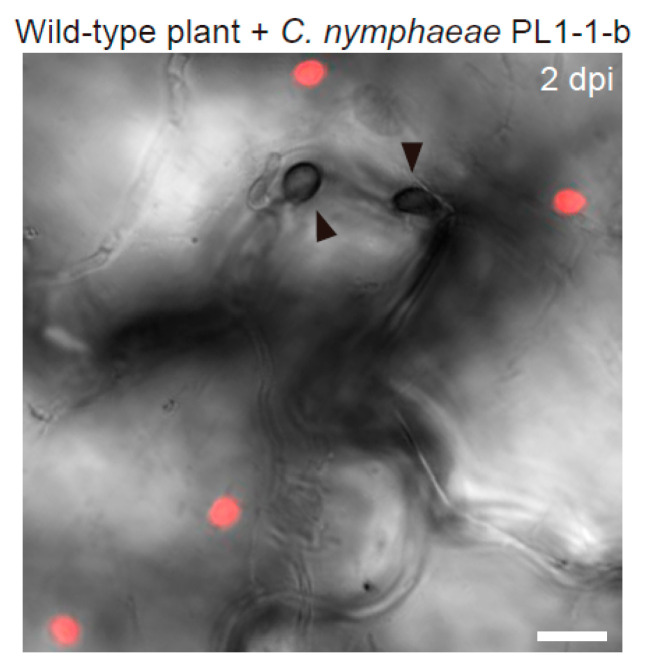
*Arabidopsis* epidermal chloroplast response (ECR) against nonadapted fungal pathogen *Colletotrichum nymphaeae* PL1-1-b. The epidermal surface of the pathogen-inoculated cotyledon of the wild-type plant was investigated at 2 days post-inoculation (dpi). The chloroplasts were visualized based on chlorophyll autofluorescence. The DIC image was captured by confocal microscopy. The arrowheads indicate melanized appressoria. Scale bar, 10 µm.

In the steady state of *A. thaliana*, epidermal chloroplasts are not observed near the upper periclinal wall (surface) of the pavement cells, because they are usually positioned at the lower periclinal (bottom) and anticlinal walls. In the recent report, I discovered that epidermal chloroplasts emerged on the surface of pavement cells in response to *Colletotrichum* fungi with melanized appressorium formation, and named this phenomenon the epidermal chloroplast response (ECR) (Figure 1) [48].

**Figure 2:**
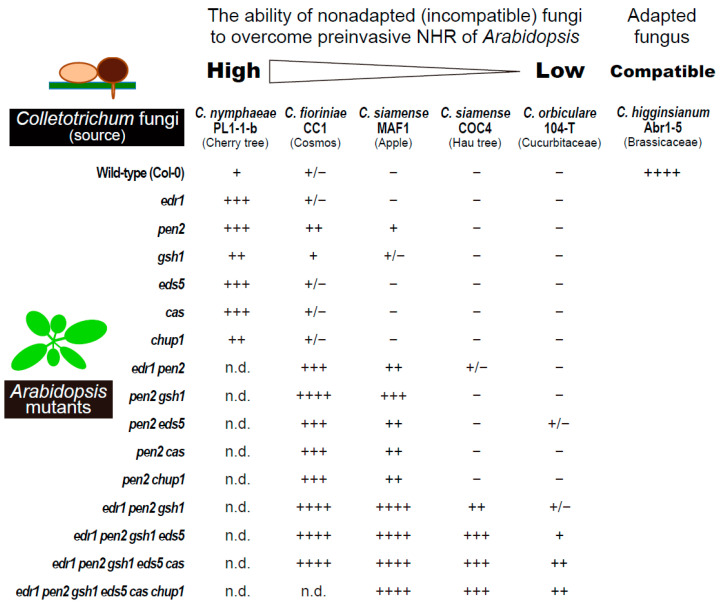
Relationships between nonadapted *Colletotrichum* fungi and preinvasive nonhost resistance (NHR) in *A. thaliana*. Invasion abilities of *C. nymphaeae* PL1-1-b (MAFF240037), *C. fioriniae* CC1 (MAFF306550), *C. siamense* MAF1 (MAFF243010), COC4 (MAFF243696), and *C. orbiculare* 104-T (MAFF240422) into the nonhost *Arabidopsis* mutants were evaluated based on the melanized appressorium-mediated entry (MAE) rates and classified. The MAE rate (%) was calculated using the following numerical formula: (the number of melanized appressoria with formation of invasive hypha)/(the number of melanized appressoria). Adapted *C. higginsianum* Abr1-5 (MAFF305635) is shown as the control. The percentage of fungal entry test as “−”, “+/−”, “+”, “++”, “+++”, and “++++” is 0–2%, 2–10%, 10–20%, 20–35%, 35–60%, and 60–100%, respectively. n.d.: not determined. Adapted from [51].

Interestingly, ECR occurs more strongly against nonadapted fungi such as the Japanese flowering cherry pathogen *Colletotrichum nymphaeae* PL1-1-b, the cosmos pathogen *Colletotrichum fioriniae* CC1, and the apple pathogen *Colletotrichum siamense* MAF1, compared to the adapted Brassicaceae pathogen *Colletotrichum higginsianum* Abr1-5, which readily penetrates and infects wild-type plants of *A. thaliana* [48]. Furthermore, the frequency of ECR varies according to the nonadapted fungal strains; the order of ECR-inducing ability in the wild-type plant is as follows: *C. nymphaeae* PL1-1-B > *C. fioriniae* CC1 > *C. siamense* MAF1, while two other nonadapted strains, hau tree pathogen *C. siamense* COC4 and cucurbit pathogen *Colletotrichum orbiculare* 104-T, do not trigger ECR in the wild-type plant [48]. Importantly, *C. siamense* COC4 and *C. orbiculare* 104-T sufficiently induce ECR in plants with mutation in the *PEN2* gene [48], which encodes an atypical myrosinase that works as a core preinvasive NHR contributor against many fungal and oomycete pathogens [6,9,13,17,48,49,50], although these two nonadapted pathogens cannot invade *pen2* mutants. The frequencies of ECR in the *pen2* mutant against *C. fioriniae* CC1 and *C. siamense* MAF1 are also higher than those in the wild-type plant, whereas other single mutants of immune-related genes such as *EDR1*, *GSH1 EDS5*, and *CAS* have little effect on ECR [48]. These results suggest that ECR is preferentially activated when PEN2-based antifungal preinvasive defense becomes ineffective in epidermal cells. Similarly, in penetration tests on multiple *Arabidopsis* mutants with many immune-related mutations, these five nonadapted *Colletotrichum* strains displayed differential ability to overcome preinvasive NHR in the same order as the ECR-inducing ability; *C. nymphaeae* PL1-1-B and *C. fioriniae* CC1 can invade the epidermis of the wild-type plant to some degree and more of the *pen2* mutant, whereas *C. siamense* MAF1, COC4, and *C. orbiculare* 104-T can break through the epidermal NHR only in the presence of *pen2* mutation or with additional mutations such as *edr1*, *gsh1*, *eds5*, and *cas* (Figure 2, Redrawn from [51]) [48,51]. Thus, there is a tight link between ECR induction in plants and the invasion ability of the nonadapted *Colletotrichum* fungi, which implies the involvement of ECR in epidermal preinvasive NHR, because these fungal pathogens are incompatible with and definitely cannot infect *A. thaliana*. The contribution of ECR to preinvasive NHR is described in a later section.

**Figure 3:**
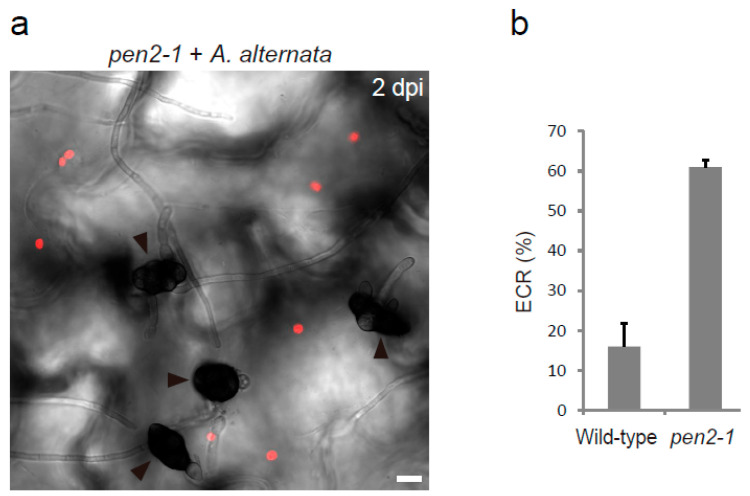
*Arabidopsis* ECR against nonadapted fungal pathogen *A. alternata* 98012501 (MAFF712212). (**a**) The epidermal surface of the pathogen-inoculated cotyledon of *pen2-1* mutant was observed at 2 dpi. The chloroplasts were visualized based on chlorophyll autofluorescence. The DIC image was captured by confocal microscopy. The arrowheads indicate melanized conidia. Scale bar, 10 µm. (**b**) Quantification of the ECR in the wild-type and *pen2-1* plants. The ratio of epidermal cells with surface chloroplasts was investigated at 2 dpi. A total of 100 cells in contact with the pathogen were observed. The means and SE were calculated from three independent plants.

ECR is not specific to *Colletotrichum.* Epidermal chloroplasts also emerge at the surface after inoculation with the nonadapted pathogen *P. oryzae*, which shows melanized appressorium-mediated entry (MAE)-type plant invasion but is phylogenetically distantly related to *Colletotrichum* [48]. The frequency of *P. oryzae*-induced ECR also increases in the *pen2* mutant. Thus, ECR may be a broad-spectrum epidermal response to a wide variety of fungal pathogens. This idea is consistent with the new finding that the nonadapted fungus *Alternaria alternata*, which is not an MAE-type pathogen and forms melanized conidia, also induces ECR in the nonhost *A. thaliana*, especially in the presence of the *pen2* mutation (Figure 3).

### 2.2. The Trigger of the Epidermal Chloroplast Response

Although how the plant epidermis recognizes fungal pathogens and induces ECR still remains an open question, some data indicate a fascinating correlation between fungal entry trial-related characteristics and ECR induction [48]. Some of the well-studied pathogen-derived molecules are pathogen/microbe-associated molecular patterns (PAMPs/MAMPs), which are highly conserved among broad-spectrum pathogens and contribute to their viability [52]. Damage-associated molecular patterns (DAMPs), host-derived molecules such as Pep1 released extracellularly during pathogen invasion, are also cues for innate immune responses [53]. PAMPs/DAMPs are recognized by plant pattern recognition receptors (PRRs), and PAMP/DAMP-triggered immunity is initiated via PRR-associated proteins, such as RLP 23 and RLP30, membrane-embedded receptor-like proteins (RLPs), FLS2 and BAK1, membrane-embedded receptor-like kinases (RLKs), and BIK1 and PBL1, receptor-like cytoplasmic kinases (RLCKs) [54,55,56,57,58,59,60,61]. Exceptional reviews on PRR signaling in plant immunity have been published by several researchers [62,63,64,65]. Intriguingly, ECR is not dependent on BAK1, BIK1, PBL1, Pep1-sensing RLKs, PEPR1 and PEPR2; therefore, PAMP and/or DAMP signaling events, at least those mediated by these RLKs and RLCKs, are not involved in the induction of ECR [48]. Furthermore, quantification of ECR in the *pen2* mutant plant against multiple *C. orbiculare* mutants deficient in different steps of invasion-related morphogenesis revealed that the formation of the penetration peg, a needle-like fungal structure emerging from the appressorium for successful entry into the plant epidermis, is essential for ECR induction [48].

Plant pathogenic fungi and oomycetes secrete an arsenal of virulence proteins, called effectors, to manipulate plant cellular processes and induce immune suppression. Numerous reviews have been published on the effectors of plant pathogens [66,67,68,69,70]. In *Colletotrichum*, fluorescently labeled effector proteins preferentially accumulate at the bottom pore of the appressorium for secretion through the penetration peg [71,72]. This effector secretion depends on the v-SNARE SEC22-mediated intracellular traffic route in the fungus [72] and, interestingly, correlates with ECR induction [48]. These observations hint at the following potential candidates as triggers of ECR: (i) pathogen-derived molecule(s) secreted from the penetration peg during fungal entry trials and (ii) plant-derived signal(s) generated in response to the degree of fungal progression in the attempt to penetrate. ECR is a universal event against many types of fungal pathogens, including *Colletotrichum* species, *P. oryzae*, and *A. alternata* (Figure 3) [48]. Therefore, typical effectors that are limited to a narrow range of fungal pathogens for their host specificity might not be a cue of ECR; rather, common cell wall-degrading enzymes or highly conserved core effectors of a broad range of pathogenic fungi or plant damage signals not associated with Pep1 might trigger ECR in plant epidermis.

### 2.3. The Regulators of Epidermal Chloroplast Response and Preinvasive Nonhost Resistance of Arabidopsis

**Figure 4:**
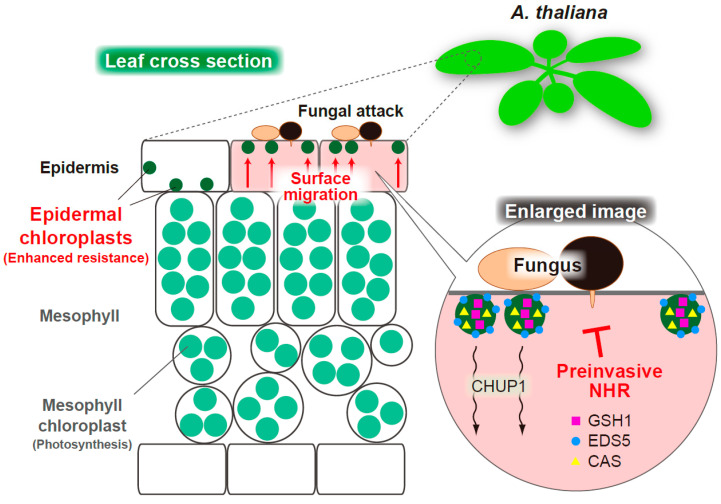
Schematic overview of the ECR-mediated preinvasive NHR in epidermis of *A. thaliana*. An MAE trial of nonadapted fungi triggers the CHUP1-related ECR. The epidermal chloroplast-localized immune components GSH1, EDS5, and CAS alter their intracellular locations on activation of ECR. The ECR and these immune components contribute to epidermal preinvasive NHR against fungal pathogens.

Large mesophyll chloroplasts show intracellular movements based on the intensity of light for safe and efficient photosynthesis; they escape from strong light and migrate to the anticlinal walls of the cell to prevent photodamage (avoidance response) and move toward weak light and settle on the periclinal walls to increase photosynthetic efficiency (accumulation response). Many regulatory proteins of this adaptive phenomenon, called chloroplast photorelocation movements, have been identified within mesophyll cells [73]. However, there are few reports and little is known about the components that control the intracellular movement of epidermal chloroplasts [74,75]. In this context, I recently demonstrated that ECR in epidermal cells shares common regulatory proteins with mesophyll chloroplast photorelocation movements [48]. The actin-binding protein CHUP1 generates a chloroplast-actin-based motive force and is essential for both accumulation and avoidance responses [76,77,78], while the auxilin-like J-domain protein JAC1 regulates the appearance and disappearance of chloroplast-actin filaments and is required for the accumulation response [79]. The genetically modified *A. thaliana* shows that overexpression of CHUP1 proteins causes strong suppression of the ECR. Typically, epidermal chloroplasts are hardly detected at the surface area, even after inoculation with nonadapted fungal pathogens [48]. However, the overexpression of JAC1 or deletion of CHUP1 leads to constitutive positioning of epidermal chloroplasts at the surface, regardless of fungal inoculation [48]. Thus, CHUP1 and JAC1 regulate negatively and positively, respectively, the positioning of epidermal chloroplasts at the surface area of pavement cells (Figure 4). It is noteworthy that the inoculation of the *chup1* mutant with high concentrations of nonadapted fungi only slightly increased the population of surface chloroplasts, thereby suggesting the impairment of ECR by the depletion of CHUP1 protein. In contrast, phototropins 1 and 2, which are blue light receptors responsible for chloroplast photorelocation movements [80,81,82], are dispensable for the activation of ECR [48]. Therefore, chloroplast photorelocation and ECR have different stimulus recognition systems for light and pathogens, respectively, but share at least some downstream regulatory components. From the viewpoint of the differences between mesophyll and epidermal chloroplasts, the versatility of the adaptive systems against different environmental stresses might reflect the elaborate survival strategy of the plant.

Whether ECR contributes to plant immunity in the epidermal cells of *A. thaliana* is a fascinating question. To clarify the involvement of ECR in preinvasive NHR, the effects of ECR impairments by overexpressing or depleting CHUP1 proteins on the MAE rate of multiple nonadapted fungi into the plant epidermis have been investigated [48]. *C. nymphaeae* PL1-1-B, which exhibited relatively lower incompatibility with *A. thaliana* compared to other nonadapted fungi, showed an increased MAE rate in ECR-impaired plants (Figure 2) [48]. Moreover, in plants with *pen2* or other additional mutations such as *edr1*, *gsh1*, *eds5*, and *cas*, an increase or decrease in the levels of CHUP1 proteins promoted the MAE rate of *C. fioriniae* CC1, *C. siamense* MAF1, COC4, and *C. orbiculare* 104-T (Figure 2) [48]. Similar results were obtained for MAE rate of *P. oryzae*. Accordingly, ECR is a stress response of the plant, by which the preinvasive NHR is enhanced in epidermal cells when the fungal pathogens attempt invasion through the penetration peg. As described, ECR is preferentially activated in the absence of a PEN2-related defense pathway. Similarly, the inoculation of nonadapted *C. fioriniae* CC1 also induced expression of many defense-related genes, such as *PAD3*, *CYP79B2*, *CYP71A13*, *PDF1.2a*, *MYB51*, *PR1*, *FRK1*, and *NHL10*, only in the case of *pen2* mutation, although the threshold for the ECR was lower than that for the expression of these genes [48]. Based on the layered structure of the plant NHR, ECR is one of the defense responses that is programmed to back up the preinvasive NHR when the pathways involved in higher-layer preinvasive defense, including PEN2-related pathways, are ineffective [48,51]. This is consistent with the relationship between *A. thaliana* and the lower incompatible nonadapted pathogen *C. nymphaeae* PL1-1-B, which partly overcomes the high-layer preinvasive defenses [51]; *C. nymphaeae* PL1-1-B strongly triggers the ECR, and also induces defense-related genes in the wild-type plant [48]. A number of phytopathogenic fungi, such as *C. nymphaeae* PL1-1-B, that partly overcome the higher-layer preinvasive defenses of *A. thaliana* is presumed to exist in nature. Therefore, plants have evolved ECR as one of the mechanisms of epidermal NHR against these kinds of fungal pathogens (Figure 4).

## 3. Epidermal Chloroplast-Localized Immune Components Contribute to Preinvasive Antifungal Nonhost Resistance of *Arabidopsis*

### 3.1. The Preferential Localization of Immune-Related Components to Motile Epidermal Chloroplasts in A. thaliana

How ECR contributes to preinvasive NHR against nonadapted fungal pathogens remains to be completely elucidated. Fluorescence live cell imaging revealed that immune-related components, such as γ-glutamylcysteine synthetase GSH1, Ca^2+^-sensing receptor CAS, MATE family transporter EDS5, isochorismate synthase ICS1/SID2, and aminotransferase ALD1, were localized in epidermal small chloroplasts (Figure 4) [15,48,83,84,85,86]. Interestingly, the fluorescent signals of these components in epidermal chloroplasts are much stronger than those in large mesophyll chloroplasts [48,83,85,86]. In ECR-activated pavement cells after inoculation with nonadapted *C. fioriniae* CC1, the GSH1, EDS5, and CAS proteins were found to localize to the surface area together with the motile epidermal chloroplasts, suggesting a tight link between ECR and the intracellular positioning of these immune components [48]. These observations could strengthen the importance of epidermal chloroplasts in plant immunity.

### 3.2. Epidermal Chloroplast-Localized Immune Components and Preinvasive Defense in A. thaliana

GSH1 contributes to plant defense via glutathione biosynthesis in both compatible and incompatible *A. thaliana*-pathogen interactions [15,87]. CAS is involved in transient Ca^2+^ signaling in chloroplasts during plant immunity [88], and the CAS-targeted fungal effector of *Sclerotinia sclerotiorum* interferes with SA signaling in the host plant [89]. ICS1/SID2 and EDS5 are required for the biosynthesis and transport of SA precursors, respectively, during the plant immune response [85,90,91]. The tight link between these immune components and epidermal chloroplasts implies their involvement in epidermal NHR against fungal pathogens that trigger ECR. The contributions of GSH1, CAS, EDS5, and ICS1/SID2 to the preinvasive NHR against nonadapted fungi have been demonstrated; *C. nymphaeae* PL1-1-B showed increased MAE in the epidermis of *gsh1*, *cas*, and *eds5* single mutants compared to that in the wild-type plant [51], whereas similar effects of these mutations on MAE of *C. fioriniae* CC1, *C. siamense* MAF1, and *P. oryzae* were observed in each double mutant with *pen2* mutation (Figure 2) [48]. The increased MAE rates of more incompatible *C. siamense* COC4 and *C. orbiculare* 104-T were confirmed in *pen2* mutant plants with multiple mutations in immune-related genes (Figure 2) [48]. Therefore, the *pen2*-dependent effects of these mutated genes, which encode epidermal chloroplast-localized proteins, correlate with ECR triggered by each fungal pathogen. This study proposes that *Arabidopsis* epidermal cells deploy an ECR-centered immune response, wherein the intracellular repositioning of immune-related proteins might enhance antifungal NHR (Figure 4) [48]. The epidermal chloroplast-specific protein, ALD1, is essential for local disease resistance and systemic acquired resistance against both virulent and avirulent bacterial pathogens, and it exerts its effect by controlling SA accumulation [86,92,93,94]. In the future, it should be elucidated whether ALD1 also functions in the preinvasive NHR against fungal pathogens.

## 4. Epidermal Chloroplasts Accumulate at the Interface with Oomycete Pathogen and CHUP1 Is Required for Penetration Resistance in *Nicotiana benthamiana*

### 4.1. Focal Accumulation of Epidermal Chloroplasts at the Interface with Oomycete Pathogen in N. benthamiana

**Figure 5:**
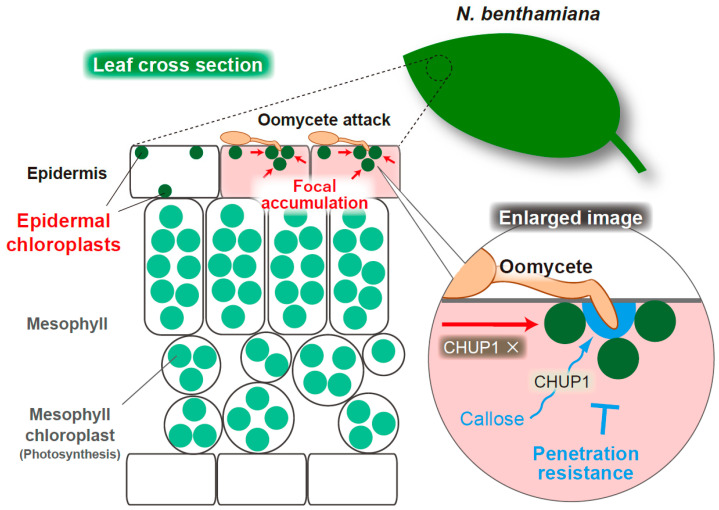
Schematic overview of the focal accumulation of the epidermal chloroplasts and callose deposition at the penetration site of oomycete *P. infestans* in epidermis of *N. benthamiana*. A hyphal entry of *P. infestans* triggers the intracellular movements of epidermal chloroplasts to the interfaces in a CHUP1-independent manner, while CHUP1-dependent callose deposition occurs at the penetration site of the pathogen and might contribute to penetration resistance.

The role of epidermal chloroplasts in response to the oomycete pathogen *P. infestans* has also been studied in the model solanaceous plant *N. benthamiana* [95,96,97]. In this section, I mention recent research on the dynamics of epidermal chloroplast movements and the involvement of CHUP1 in antioomycete immunity, although there is ambiguity regarding the adaptive or nonadaptive characteristics of *P. infestans* to *N. benthamiana*, which shows an ambiguous age-related resistance to *P. infestans*; mature plants are resistant, whereas young plants are susceptible [98]. During the response of *N. benthamiana* to *P. infestans*, epidermal chloroplasts show intracellular movements and accumulate at the interface with the pathogen haustorium, an infection-related specialized hyphae, in a BAK1-independent manner (Figure 5) [96]. Thus, focal immunity mediated by epidermal chloroplasts has been proposed in *N. benthamiana-P. infestans* pathosystem. A similar phenomenon was observed in the ECR-activated cells of *A. thaliana* during the antifungal response; however, most of the surface chloroplasts were scattered in the case of ECR and, at least under the microscope, did not associate with the pathogen interface [48]. Given that epidermal chloroplasts of *N. benthamiana* are usually detected at the surface area of pavement cells, regardless of pathogen inoculation, the working mechanisms of epidermal chloroplasts in response to pathogen attacks were not entirely the same between *A. thaliana* and *N. benthamiana*. Furthermore, it is possible that the CHUP1-related ECR and focal accumulation of epidermal chloroplasts to the fungal penetration sites are independent events in *A. thaliana* because the pathogen interface-specific accumulation of epidermal chloroplasts in *N. benthamiana* is CHUP1-independent (Figure 5) [97]. In this context, it remains to be elucidated whether a part of the epidermal chloroplasts of *A. thaliana*, which focally accumulate at the fungal penetration sites, has a distinct role(s) from ECR-associated epidermal chloroplasts, as well as that in *N. benthamiana*.

### 4.2. CHUP1-Dependent Callose Deposition at the Oomycete Penetration Site during the Epidermal Resistance of N. benthamiana

Savage et al. reported that the positioning of epidermal chloroplasts at the interface between *N. benthamiana* and the haustoria of *P. infestans* occurred independent of CHUP1 [97], which was slightly different from *Arabidopsis* ECR [48]. However, they demonstrated that CHUP1 is required for penetration resistance in epidermal cells of *N. benthamiana* against *P. infestans* [97]. Silencing or knockout of two *CHUP1* alleles in *N. benthamiana* clearly enhances susceptibility to *P. infestans*, whereas the accumulation of epidermal chloroplasts around haustoria is not impaired in the *CHUP1* knockout mutant (*chup1*) [97]. The *Arabidopsis chup1* mutant also showed decreased preinvasive NHR against MAE of nonadapted fungal pathogens [48], suggesting the importance of the chloroplast-associated protein CHUP1 in epidermal plant immunity against both oomycete and fungal pathogens.

Interestingly, focal callose deposition at the haustoria of *P. infestans*, which is one of the defense responses of the plant, is CHUP1-dependent, suggesting that the chloroplast-associated protein CHUP1 contributes to epidermal immunity against oomycete pathogen by enhancing penetration resistance via callose deposition (Figure 5) [97]. The key question is how the link between this callose deposition and the focal accumulation of the epidermal chloroplasts at the pathogen interface exists, because CHUP1 is a chloroplast-associated protein. In this regard, Savage et al. showed no correlation between callose deposition and the presence of chloroplasts around the haustorium [97]. Consistent with this finding, in *A. thaliana*-*C. orbiculare* incompatible interaction, callose deposition does not correlate with ECR activation; the callose is sufficiently deposited at the sites of pathogen penetration attempts in the wild-type plant, where the ECR is hardly activated due to a prior function of high-layer preinvasive defense(s) [48]. *CHUP1* gene knockout in *N. benthamiana* has no effect on other core immune processes, such as PAMP-triggered MAPK phosphorylation, constitutively active MEK2^DD^-induced hypersensitive response (HR)-like cell death, and effector-triggered HR cell death [97]. Therefore, Savage et al. speculated that in the *CHUP1*-knockout *N. benthamiana*, reduction in the production of epidermal chloroplast-derived molecules, such as ROS and SA precursors, could plausibly cause the impairment of callose accumulation around the haustorium. They also did not exclude the possibility that snapshot imaging did not accurately reflect the involvement of epidermal chloroplast mobilization to the haustoria in focal callose deposition, because the chloroplasts may have moved away from the haustoria after callose deposition [97]. I expect future research to clarify the regulatory mechanism of callose deposition at the pathogen interface via chloroplast-associated CHUP1 in *N. benthamiana*, and compare it with the epidermal chloroplast-related immune responses in *A. thaliana*.

## 5. Dynamic Morphology of Epidermal Chloroplasts and Inter-Organelle Interactions in Plant Immunity

### 5.1. Stromule Formation, Enlargement, and Cavity Formation

Mesophyll and epidermal chloroplasts grow dynamic tubular extensions called stromules, which are filled with stroma [99]. In epidermal cells of *N. benthamiana*, stromules increase during antiviral and antibacterial immunities, where effector-triggered HR cell death occurs [95]. *N. benthamiana* also induces stromule formation during PAMP-triggered immunity against the oomycete INF1 in addition to bacterial flg22 and fungal chitin in a BAK1-dependent manner [95,96]. Interestingly, stromule induction is suppressed by the *P. infestans* effector AVR3a [96]. Stromule induction in epidermal chloroplasts was also observed during antibacterial immunity in *A. thaliana* [95]. Altogether, these observations imply the possible involvement of stromules in plant defense responses. Importantly, chloroplast movements and connections with other organelles, such as nuclei, are mediated by stromules during plant immune response [95,100]. Moreover, stromules may transport the defense-related signaling molecule ROS and the chloroplast defense protein NRIP1 to the nucleus in *N. benthamiana* [95,101]. NRIP1 is required for antiviral resistance in *N. benthamiana* [102]. Immune-related proteins GSH1, EDS5, and CAS are also localized to stromules extending from the epidermal chloroplasts in *A. thaliana*, although the localization signal of the thylakoid protein CAS in stromules is weaker than that of the other two proteins and may possibly be an artifact of overexpression [48]. Thus, in epidermal cells, immune-related components can dynamically alter their positioning through chloroplast movements and further expand their location to the outside of the chloroplasts via stromules during immune responses. However, it is also highly controversial as to whether increased stromules have positive effects on antimicrobial resistance. Caplan et al. reported that *CHUP1* gene-silenced *N. benthamiana* and *Arabidopsis chup1* mutants showed increased stromules, which were visualized with NRIP1-GFP fusion protein, in the absence of pathogens and promoted effector-triggered HR cell death during antiviral and antibacterial immune response [95]. In contrast, Savage et al. demonstrated that *chup1* mutants of both *N. benthamiana* and *A. thaliana* exhibited decreased stromules, which were visualized with GFP, in the absence of pathogens [97]. Moreover, the *chup1* mutant of *N. benthamiana* can induce stromule formation sufficiently when challenged with *P. infestans*, despite its increased susceptibility to this pathogen [97]. Decreased preinvasive NHR in *Arabidopsis* with *a chup1* mutation against MAE-type fungal pathogens has also been reported [48]. These discrepancies can be explained in future studies. It is noteworthy that, in plant immunity, stromule induction and chloroplast movements, such as ECR and focal accumulation at the pathogen interface, are more related but clearly different events, because the former depends on the PAMP signaling kinase BAK1, while the latter does not. Therefore, distinct pathogen-derived signals may be integrated through an epidermal chloroplast-centered system to harmonize defense responses.

**Figure 6:**
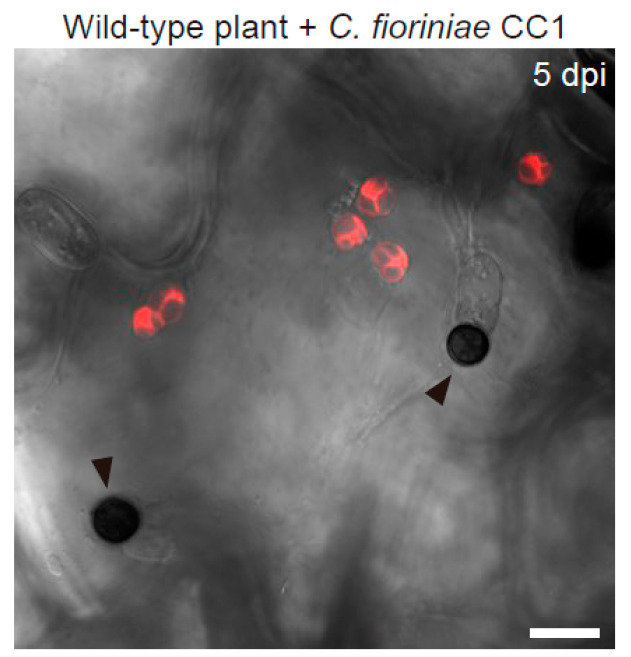
Enlargement of epidermal chloroplasts during ECR in *A. thaliana*. The epidermal surface of the wild-type plant inoculated with nonadapted *C. fioriniae* CC1 was investigated at 5 dpi. The chloroplasts were visualized based on chlorophyll autofluorescence. The DIC image was captured by confocal microscopy. The arrowheads indicate melanized appressoria. Scale bar, 10 µm.

The epidermal chloroplasts are considerably smaller than the mesophyll chloroplasts. However, intriguingly, enlarged epidermal chloroplasts in ECR-activated cells could be observed, and the population of the enlarged chloroplasts gradually increased during incubation with the nonadapted fungal pathogens (Figure 6). Enlarged chloroplasts were not observed in the early stage of the ECR. The formation of cavities inside the enlarged epidermal chloroplasts was confirmed (Figure 6). The physiological significance of these changes in the morphology of the epidermal chloroplasts during ECR is currently unclear, but it is possible that epidermal chloroplasts enhance the preinvasive NHR via these additional events at the late stage of the ECR. Alternatively, plant organellar morphology might be affected by pathogen effectors, because it has been reported that transient expression of *Colletotrichum* effector proteins leads to enlargement of nuclei in epidermal cells of *N. benthamiana* [103]. If such organellar expansions are among the pathogen infection strategies via effectors, it is consistent with the fact that many pathogen effectors enter chloroplasts and target chloroplast-localized proteins [46,47].

### 5.2. Perinuclear Clustering of Epidermal Chloroplasts and Nuclear Movements

Chloroplasts are known to cluster around the nucleus for inter-organelle communication, where the transmission of retrograde signals occurs in response to various environmental stresses [37,38,104,105,106]. In the defense responses of *N. benthamiana*, retrograde ROS signaling from perinuclear clustering chloroplasts to the nucleus through stromules has been reported [95]; the same has been proposed in *A. thaliana* [88,107,108]. Interestingly, the loss of the *CHUP1* gene leads to increased perinuclear clustering of epidermal chloroplasts in both *N. bnethamiana* and *A. thaliana* [97]. Perinuclear clustering of epidermal chloroplasts was also observed during *Arabidopsis* ECR after pathogen inoculation or in CHUP1-depleted conditions with no pathogen [48]. Thus, these two plant species have similar regulatory mechanisms to control perinuclear clustering of epidermal chloroplasts, in which CHUP1 shows negative effects. Given the impairment of the ECR and preinvasive NHR by the constitutive surface positioning of epidermal chloroplasts in the *chup1* mutant of *A. thaliana* [48], greater perinuclear clustering of epidermal chloroplasts might also be functionally deteriorated. Consistent with this idea, *CHUP1*-knockout *N. benthamiana* is more susceptible to *P. infestans* [97].

Light-induced nuclear movement in *Arabidopsis* mesophyll and epidermal pavement cells is CHUP1-dependent [74,78]. CHUP1 specifically localizes to the envelope of chloroplasts, but not nuclei, and regulates chloroplast movements; hence, the CHUP1-mediated intracellular movement of epidermal chloroplasts is a motive force for nuclear movement in response to strong light [74,78]. Similarly, nuclei, together with perinuclear clustering chloroplasts, migrate to the surface area during ECR in *A. thaliana*, which is tightly linked to the amount of CHUP1 proteins [48]. The perinuclear endoplasmic reticulum also repositions to the epidermal surface during ECR [48]. In *N. benthamiana*, the nucleus moves to the penetration sites of the oomycete *P. infestans* [96,109]. This is also observed in other plant pathosystems using fungal and oomycete pathogens [110,111,112]. The focal accumulation of the nucleus was not influenced by increased perinuclear clustering of epidermal chloroplasts in *CHUP1*-knockout *N. benthamiana* [97]. Given that the overexpression and depletion of CHUP1 proteins have opposite effects on epidermal chloroplast movements during *Arabidopsis* ECR, it would be interesting to determine whether perinuclear clustering of epidermal chloroplasts is suppressed by CHUP1 overexpression in *N. benthamiana*, and hence whether the focal accumulation of the nucleus at the *P. infestans* interface is perturbed.

## 6. Conclusions

The importance of chloroplast function in plant immunity is widely recognized. Research focusing on the types of chloroplast has now been emerging in the field of antimicrobial responses. Given the function of the plant epidermis as a primary stronghold to repel the enemy, such as fungal and oomycete pathogens with direct penetration ability, epidermal atypical chloroplasts are an ideal research subject because of their small amount of information compared to well-known typical mesophyll chloroplasts. In particular, recent studies have revealed a link between the intracellular dynamics of epidermal chloroplasts and plant epidermal immunity, wherein the ECR, focal accumulation of epidermal chloroplasts to the pathogen interface, stromule formation, inter-organelle communication, and other processes might be orchestrated to enhance antimicrobial resistance [48,95,96,97,113]. Chloroplast-localized CHUP1, which was originally identified as a regulator of chloroplast photorelocation movements in mesophyll cells [76], has a key function in epidermal chloroplast-related events in response to pathogens. JAC1, a regulator of the photoinduced accumulation response of chloroplasts, is also involved in the intracellular positioning of epidermal chloroplasts during ECR [48]. To understand the mechanism underlying epidermal immunity via motile chloroplast-centered responses, further analysis is needed on which and how the components employed in the chloroplast photorelocation system contribute to the defense responses in plant epidermis.
